# A Decomposition of Life Expectancy and Life Disparity: Comparison Between Hong Kong and Japan

**DOI:** 10.15171/ijhpm.2019.142

**Published:** 2020-01-27

**Authors:** Yan Zheng, Mengni Chen, Paul SF Yip

**Affiliations:** ^1^Department of Social Work and Social Administration, Faculty of Social Sciences, The University of Hong Kong, Hong Kong, China.; ^2^Center for Demographic Research, Université catholique de Louvain, Ottignies-Louvainla-Neuve, Belgium.; ^3^HKJC Centre for Suicide Research and Prevention, The University of Hong Kong, Hong Kong, China.

**Keywords:** Mortality, Life Expectancy, Life Disparity, Hong Kong, Japan

## Abstract

**Background:** Life expectancy and life disparity are 2 useful indicators to assess the health condition of a society. Both Hong Kong and Japan have one of the longest life expectancies in the world. Recently, Hong Kong has overtaken Japan and topped the life expectancy rankings. However, whether Hong Kong has also outperformed Japan in life disparity is still unknown.

**Methods:** Decomposition analyses have been conducted to evaluate age-specific contributions to the changes in life expectancy and life disparity for each of the populations. Furthermore, the differences between the 2 populations were examined over the period 1977-2016.

**Results:** Reduction in mortality of the adult and the old age groups contributes most to the increase in life expectancy for the study period. Hong Kong has a higher life disparity than Japan, and due to the great improvement in reducing premature deaths, the Hong Kong-Japan gap has been narrowing. However, in recent years, further reduction in mortality of the oldest elderly in Hong Kong has actually contributed to the increase in its disparity, thus widening its gap with Japan again.

**Conclusion:** Increasing dominant influence of "saving lives at late ages" is very likely to cause the reemergence of increasing life disparity in these 2 long-lived populations.

## Background

Over the last several decades, life expectancy throughout the world has been rising substantially. Life expectancy is a widely used summary indicator of the mortality profile of a population. It reflects the overall health of a society^1^ and often serves as an early predictor of societal issues.^2^ Therefore, research on life expectancy is of great importance in assessing health status and evaluating effectiveness of health policies.

However, while celebrating the achievement in prolonging longevity, another important question is “whether the increasing length of life is enjoyed equally.” That is, with the increase in life expectancy, whether the inequality in the length of life has been reduced? The difference in age at death reflects inequality in health, and some scholars have argued that it is more straightly connected with absolute deprivation than inequality of income, education or occupational attainment,^1^ and is thus called the “final inequality.”^3^

To reflect the variations and inequality of lifespans, several measures such as the standard deviation in age at death for ages 10 and older (S_10_),^4^ the interquartile range,^5^ the Gini coefficient,^6^ and the Theil index of inequality^1^ have been proposed. In this paper, the interest is in life disparity at birth (e_0_^†^), which is defined as the average life years lost due to death.^7^ This indicator, which was highlighted by Vaupel and Romo,^8^ has been increasingly used to measure the dispersion of age at death and reflect how much lifespans vary among individuals. Greater life disparity means greater lifespan variations and higher uncertainty in the age at death. However, it should be noted that whether life disparity increases or decreases depends on the balance between “saving lives at early ages” and “saving lives at late ages.”^7^ Zhang and Vaupel have identified a threshold age (*a*^†^): reduction in mortality before that age leads to lower life disparity, while reduction in mortality after that age results in higher life disparity.^9^ Hence, an age-specific investigation of changes in life expectancy and disparity is important to understand the underlying mortality dynamics.

Does greater longevity come with lower life disparity? After investigating the life expectancy and disparity in 40 countries and regions, Vaupel and colleagues have found that “several life expectancy leaders were also top life disparity leaders,” meaning that countries which enjoy long life expectancy also enjoy egalitarian lifespan distribution. Japanese females are remarkably successful in both dimensions.^7^ Hong Kong, a special administration region of China, however, with a 7.5 million population^10^ and one of the longest-lived societies in the world, was omitted from their analyses. Recently, Hong Kong has overtaken Japan, and topped the life expectancy ranking in the world. How is Hong Kong compared with Japan in life disparity, or has Hong Kong achieved the longest life expectancy at the expense of life disparity?

Over the past four decades, life expectancy in Hong Kong has experienced a very impressive rise from 70.1 years in 1977 to 81.3 years in 2016 for males, and from 76.7 years to 87.3 years for females.^10^ Previous studies have well documented the improvement in life expectancy in Hong Kong.^11-14^ These studies have enhanced the understanding of the health conditions of Hong Kong residents. However, the life disparity in Hong Kong has thus far not been examined. Hong Kong and Japan are high-income Asian societies with longest life expectancies and ultra-low fertility rates in the world.^15,16^ Although they are different in population size, the 2 societies face similar demographic challenges – low fertility and long life expectancy resulting in a rapidly ageing society. In comparison to many other developed countries, as Japan has achieved very high life expectancy and very low life disparity, taking Japan as a reference when investigating the life disparity in Hong Kong would help to understand its average health condition at a societal level from a more global perspective. The results should be of interest and are also relevant to other Asian high-income societies such as South Korea and Singapore.

In this study, both the life expectancy and life disparity in Hong Kong are investigated and compared with Japan over the past 40 years. The goals are (1) to find out the common drivers that make the 2 Asian societies achieve the longest life expectancies; (2) to demonstrate the detailed patterns of life disparity that underlie the increasing longevity; and (3) to reveal that notwithstanding similar life expectancies, how life disparity differs between Hong Kong and Japan. To achieve the goals, decomposition analyses have been conducted to quantify the age-specific impacts on the changes of the 2 indicators during the period 1977-2016 for Hong Kong and Japan. Furthermore, the differences in life expectancy and life disparity between the 2 societies have been examined.

This paper is structured as follows: a brief description of the sources of the data and the decomposition method are provided in the Methods section; the trends in age-specific mortality rates, life expectancy, and life disparity over the period 1977-2016 are displayed in the Results section. Then, the results of decomposing the changes in life expectancy and disparity over time as well as the comparisons between Hong Kong and Japan in life expectancy and disparity are presented. Discussion and Conclusion are given in the final section.

## Methods

For the decomposition analyses, mortality data and life tables over the period 1977-2016 for Hong Kong and Japan are required. The data for Japan were from the Human Mortality Database (HMD).^17^ The HMD provides carefully checked life tables for many countries,^18^ which has been increasingly used for research of human longevity. In the HMD, the open age category is 110 and above, and the death rates for ages 80 to 110+ were smoothed by fitting the Kannisto model.^19^ In this study, the open age category of Japan was transformed to ages 100 and above so as to improve on the comparison with Hong Kong.

The data for Hong Kong were from the Census and Statistics Department, which provides life tables of good quality, covering the period 1971-2018 (Table E484: Hong Kong Life Tables, 1971-2018, from https://ww1.censtatd.gov.hk/hkstat/sub/sp190.jsp?productCode=D5320184). The life tables of Hong Kong are compiled based on actual mortality data. Graduation techniques, eg, Beer’s modified formula, are used to eliminate the random fluctuation in the deaths rates. For higher old ages, Coale-Kisker method, for instance, is adopted for smoothing, which assumes a decrease in the rate of increase in mortality at high ages.

Since different smoothing methods are used for the 2 populations, additional check has been performed to test whether the results are still consistent by applying Kannisto model to Hong Kong’s mortality rates among old people aged 80 and above. It turns out that there is no significant change in the results. Therefore, the life tables compiled by Hong Kong Census and Statistics Department are directly used for analyses.

Life expectancy and life disparity at birth for both Japan and Hong Kong were analyzed. As an aggregated demographic measure, life disparity has a crucial public health interpretation^20^ and can be additively decomposable.^21^ Besides, life disparity is proved highly correlated with other indicators of lifespan variation, thus it is reasonable to take it as a substitute for other indicators.^7,9^ For empirical calculation, the following formula can be used:

$$e_{0}^{†}=\frac{1}{l_{0}}\sum_{x=0}d_{x}(e_{x}+a_{x}(e_{x+1}-e_{x}))+\frac{1}{l_{0}}l_{w}*e_{n}$$

where *l*_0_ is the number of survivors at age 0; *d*_x_ is the number of deaths between age *x* and age *x+*1; *e*_x+1_ is the expectation of life at age* x+*1; and *w* is the limiting age *a*_x_ is the average number of years lived between age *x* and *x+*1. For age 0, *a*_x_ is estimated based on the method of Andreev and Kingkade which has also been adopted by HMD.^19^ For other age groups, the value is assumed to be 0.5. In terms of the open age category, *a*_100+_= 1/* m*_100+_ (*m*_x_ is the mortality rate at age *x*, and *m*_100+_ is the mortality rate of those aged 100 and over).

Unlike the improvement in life expectancy, which can be achieved by saving lives at any ages, the reduction in life disparity is determined by the balance between averting deaths at early ages and averting deaths at late ages, which is separated by the threshold age (*a*^†^). Saving lives before the threshold age would reduce the disparity, while saving lives after the threshold age would increase the disparity.^7^ Threshold age, *a*^†^ can be obtained by finding the age at which *e*^†^(*a*) = *e*(*a*) (1 – *H*(*a*)) with interpolation method, where (with *μ*(*x*) denoting the age specific hazard of death) is the cumulative hazard function.^9^

In this study, firstly, a decomposition analysis is performed to estimate the age-specific contributions to the changes in life expectancy and disparity over the period 1977-2016 in Japan and Hong Kong. Then, age-specific contributions to the differences in life expectancy and disparity between Hong Kong and Japan are estimated. The decomposition method was proposed and used by Shkolnikov and Andreev.^22^ By using mortality data of Hong Kong from HMD that have been recently released publicly online for the period 1986-2017, a robustness check was conducted to further justify our results (see Supplementary file 1).

## Results

### Mortality, Life Expectancy, and Life Disparity During the Period 1977-2016

**Figure 1:**
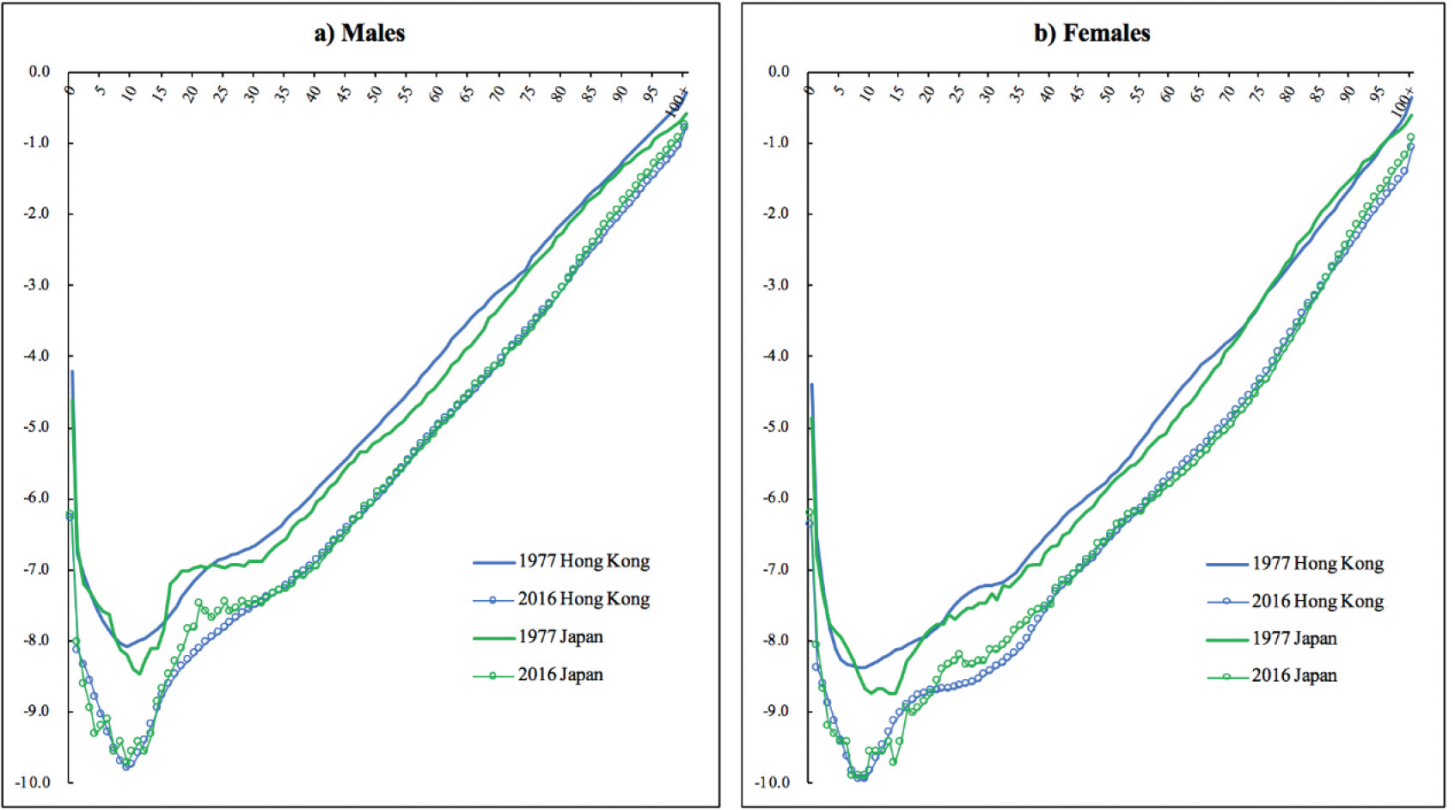
The Logarithm of Age-Specific Mortality Rates in 1977 and 2016: Hong Kong and Japan. Note: For both Hong Kong and Japan, the mortality rates are smoothed estimates from the sources of death data.

Figure 1 shows the patterns of age-specific mortality rates for males and females in Hong Kong and Japan. Both 2 societies have experienced significant reductions in mortality rates during the observed period. In 1977, the male mortality rates in Hong Kong were higher than Japan across almost all ages, and the female mortality rates were also higher than Japan. Over the past 40 years, the gap in mortality rates between Hong Kong and Japan has narrowed, or almost closed for both genders. In 2016, the mortality rates for the majority of the age groups in Hong Kong seemed to be at the same levels as Japan. It should further be noted that male mortality rates at advanced ages in Hong Kong had a better performance in 2016, while the old females maintained this advantage consistently during the observed years.

**Figure 2:**
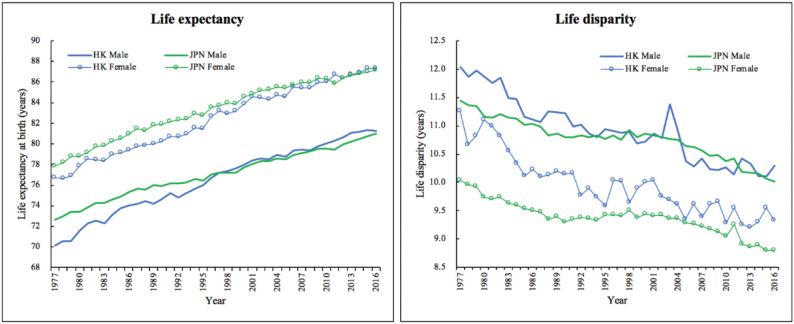
Trends in Life Expectancy and Life Disparity at Birth in Hong Kong and Japan During the Period 1977-2016.

Figure 2 clearly presents the time trends in life expectancy at birth (*e*_0_) and life disparity at birth (*e*_0_^†^) in Hong Kong and Japan from 1977 to 2016. Apparently, male and female life expectancies in both Hong Kong and Japan have experienced continuous increases. In 1977, the male life expectancy in Hong Kong was lower than Japan by about 2.6 years; nonetheless, in the ensuing 20 years, it has caught up, and finally overtook Japan in 1998, since then, it has maintained the leading position. The female life expectancy in Japan was higher than Hong Kong for a long period until 2011 when Hong Kong has also finally exceeded Japan.

With regard to life disparity, in both Hong Kong and Japan, the life disparity for males is always higher than females; and over the period under study, the life disparities for both genders have been declining in the 2 societies. The gap in male life disparity between Hong Kong and Japan has been diminishing; even in the late 2000s, the life disparity in Hong Kong was lower than Japan. Compared with males, the gap in female life disparity between the 2 places is relatively larger.

**Figure 3:**
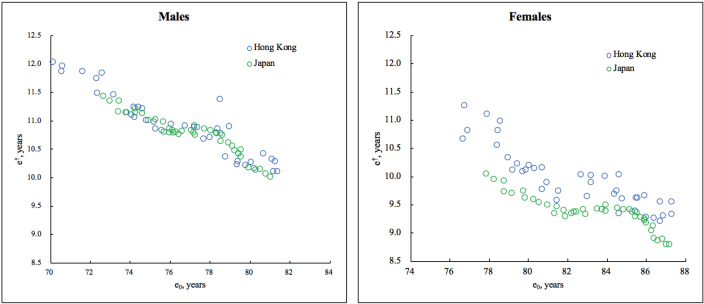
The Relationship Between Life Expectancy and Life Disparity at Birth in Hong Kong and Japan During the Period 1977-2016.

Figure 3 demonstrates a generally negative correlation between life expectancy and life disparity in Hong Kong and Japan during the period 1977-2016. Although Hong Kong males started with a lower life expectancy and a higher life disparity, over a long period, the trajectories for Hong Kong and Japan converged. On the contrary, as the life expectancy increased in earlier decades, the female life disparity in Hong Kong declined at a steeper speed, while in later years, compared with Japan, despite the closeness in life expectancy, the female life disparity in Hong Kong was still greater.

**Figure 4:**
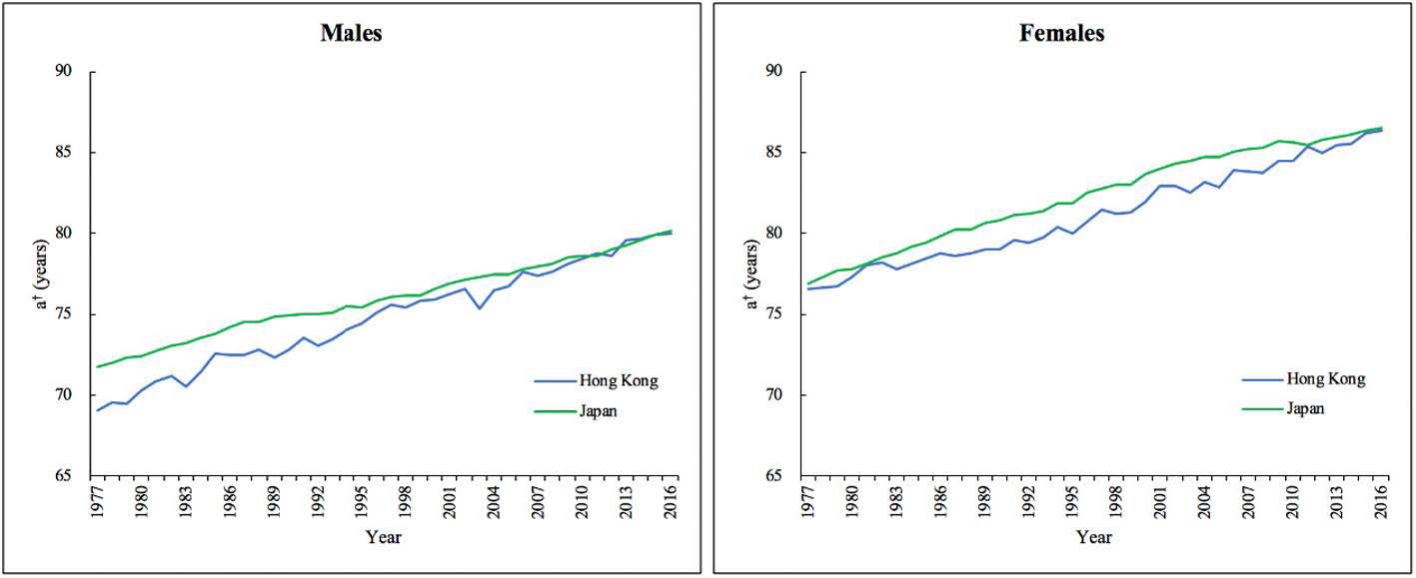
Trends in Threshold Ages (a^†^) in Hong Kong and Japan in 1977-2016.

Figure 4 shows the trends in threshold ages in Hong Kong and Japan over the period 1977-2016. For most of the observed years, Japan has higher threshold ages than Hong Kong for both 2 genders. However, in recent years, Hong Kong caught up with Japan. In 2016, the threshold ages of males in the 2 societies were about 80 years, which meant that reducing mortality before age 80 would lead to the decline in life disparity while reducing mortality after age 80 would increase life disparity. The threshold ages of females were about 86, indicating that the decline in the mortality rates among the oldest elderly would enlarge the disparity of females.

### Decomposition of Life Expectancy and Life Disparity Over Time

Table 1 presents the decomposition of changes in the male and female life expectancy over the period 1977-2016 in Hong Kong and Japan. Compared with Japan, Hong Kong has a larger increase in life expectancy of 11.15 years (about 15.9% increase from 1977) for males and 10.56 years (about 13.8% increase from 1977) for females. It was evident that the declining mortality at the adult ages (25-59 years) and the old age groups (60-74 and 75-84 years) was the common driver, which explained a significant portion of the observed improvement in male life expectancy in both Hong Kong and Japan. For females, the contributions from theelderly groups (aged 60 and above) played more significant roles, with the older females aged above 85 years contributing much more remarkably than males. Compared with the adult and the old age groups, in both Hong Kong and Japan, contributions from the infants, children and teenagers, however, were relatively smaller. Specifically, the reduction in mortality rates of the 0 and 1-24 age groups has only led to about 0.5%-1.5% increase in the life expectancy of the 2 populations.

**Table 1:** Decomposition of Changes in Life Expectancy for Males and Females in Hong Kong and Japan During the Period 1977-2016

**Age Groups**	**Males**				**Females**			
	**Hong Kong**		**Japan**		**Hong Kong**		**Japan**	
	**Years**	**Percent**	**Years**	**Percent**	**Years**	**Percent**	**Years**	**Percent**
0	0.98	1.4	0.60	0.8	0.87	1.1	0.47	0.6
1-24	0.61	0.9	0.62	0.9	0.45	0.6	0.38	0.5
25-59	2.97	4.2	2.02	2.8	1.92	2.5	1.29	1.7
60-74	3.66	5.2	2.53	3.5	2.99	3.9	2.48	3.2
75-84	2.08	3.0	1.91	2.6	2.50	3.3	2.89	3.7
85+	0.84	1.2	0.65	0.9	1.82	2.4	1.80	2.3
Life expectancy in 1977	70.12		72.68		76.74		77.86	
Life expectancy in 2016	81.27		81.01		87.30		87.17	
Total Change	11.15	15.9	8.33	11.5	10.56	13.8	9.31	12.0

Note: The percentage value is calculated relative to the life expectancy in 1977.

Table 2 shows the decomposition results of the changes in life disparity in the period under study. Again, Hong Kong has a larger reduction in life disparity of 1.75 years (a decrease of about 14.5% from 1977) for males and 1.93 years (a decrease of about 17.1% from 1977) for females in the period 1977-2016. It is noteworthy that the age-specific contribution pattern of life disparity is quite different from that of life expectancy: for both genders, the younger age groups and the older age groups have quite opposite impacts on life disparity in the 2 societies.

**Table 2:** Decomposition of Changes in Life Disparity for Males and Females in Hong Kong and Japan During the Period 1977-2016

**Age Groups**	**Males**				**Females**			
	**Hong Kong**		**Japan**		**Hong Kong**		**Japan**	
	**Years**	**Percent**	**Years**	**Percent**	**Years**	**Percent**	**Years**	**Percent**
0	-0.83	-6.9	-0.52	-4.5	-0.76	-6.7	-0.42	-4.1
1-24	-0.50	-4.2	-0.52	-4.5	-0.39	-3.4	-0.33	-3.3
25-59	-1.82	-15.1	-1.31	-11.5	-1.36	-12.1	-0.95	-9.4
60-74	-0.77	-6.4	-0.66	-5.8	-1.31	-11.7	-1.16	-11.5
75-84	0.90	7.5	0.66	5.7	0.01	0.1	-0.03	-0.3
85+	1.28	10.6	0.93	8.2	1.89	16.7	1.64	16.3
Life disparity in 1977	12.04		11.44		11.26		10.05	
Life disparity in 2016	10.29		10.02		9.33		8.79	
Total Change	-1.75	-14.5	-1.42	-12.4	-1.93	-17.1	-1.26	-12.5

Note: The percentage value is calculated relative to the life disparity in 1977.

Specifically, in Hong Kong and Japan, for males, the reduction in mortality among the age groups of 0-74 has greatly reduced life disparity, while the reduction in mortality among the age groups of 75 and above has significantly raised life disparity. The impact from the former overwhelmed the latter, thus leading to a net decline in life disparity. For females, the life disparity was reduced because of the mortality decline in ages 0-84; however, it was the age group of 85 and above that contributed conversely. Particularly, without considering the impacts from other age groups, the contribution from the age group of 85 and above would have raised the life disparity by 16.7% in Hong Kong and 16.3% in Japan. These findings have provided strong empirical evidence in support of the argument by Zhang and Vaupel that further reduction in mortality at late ages (over the threshold ages) can enlarge life disparity.^9^ Tables 1 and 2 imply that reduction in mortality of the oldest old on one hand will lead to the increase in longevity, on the other hand, it is also likely to bring about higher disparity in the future.

### Cross-Population Decomposition of Life Expectancy

**Figure 5:**
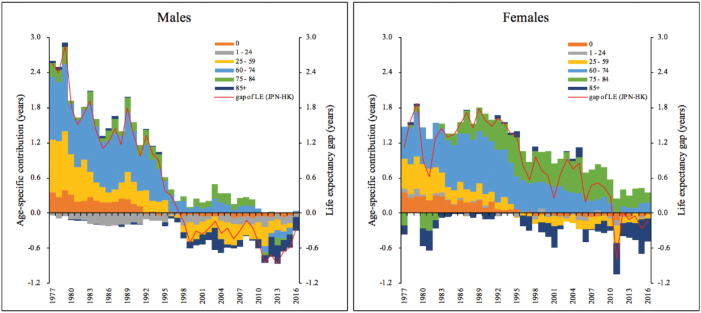
Decomposition of the Gaps in Life Expectancy Between Japan and Hong Kong During the Period 1977-2016. Note: red lines show the differences in life expectancy between Hong Kong and Japan. Abbreviation: JPN-HK, Japan minus Hong Kong.

Figure 5 visualizes the age-specific decomposition of the differences in life expectancy between Hong Kong and Japan for both males and females. The red lines show the differences in life expectancy (ie, Japan minus Hong Kong, abbreviated as JPN-HK).

As shown in early years, gaps of the male were larger than the female, but the former closed much faster than the latter. It was apparent that the advantage Japan gained in male life expectancy in the earlier decades was mainly due to lower mortality rates in the age groups of 25-59 and 60-74. Since 1998, Hong Kong has overtaken Japan in male life expectancy, this is mainly because Hong Kong has caught up with Japan in mortality at the 60-74 age group and outperformed Japan in reducing the mortality at the 25-59 and 85+ age groups. It should also be noted that for most years, males aged 75-84 contributed positively while those aged 1-24 contributed negatively to the JPN-HK gap. This means that consistently, Hong Kong had higher mortality in the former age group but lower mortality in the latter.

With regard to the gaps of females, in the first few years, Japan had longer life expectancy than Hong Kong, this was because except for the 2 oldest groups, Japan had lower mortality at almost all other age groups. As Hong Kong’s female mortality in the 0-74 age groups gradually improved to the level of Japan, the JPN-HK gap narrowed. Currently, Hong Kong’s advantage in female mortality at the oldest group (85+ years) has offset Japan’s advantage in female mortality at the 60-84 age groups, thus resulting in the absence of the gaps. It is also noteworthy that for both males and females, the contribution from Hong Kong’s dramatic decline in the mortality rates among the oldest elderly (85+ years) was very impressive.

### Cross-Population Decomposition of Life Disparity

**Figure 6:**
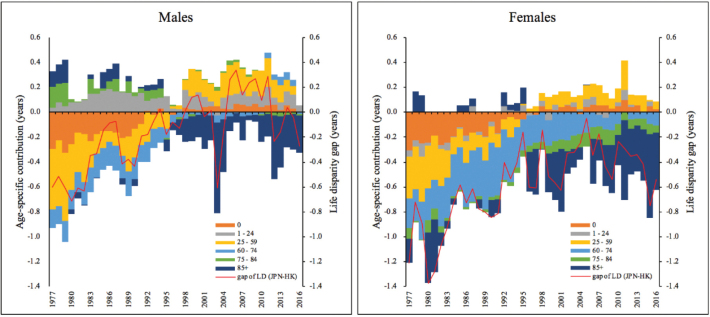
Decomposition of the Gaps in Life Disparity Between Japan and Hong Kong During the Period 1977-2016. Note: red lines show the differences in life disparity between Hong Kong and Japan. Abbreviation: JPN-HK, Japan minus Hong Kong.

Figure 6 presents the age-specific decomposition of the differences between Hong Kong and Japan in life disparity. Unlike the increase in life expectancy which can be achieved by saving lives at all ages, the change in life disparity is determined by the balance of changes in mortality at young and old age groups.^20,21^

As shown, Hong Kong had higher life disparities than Japan for both males and females in the earlier periods. This was mainly contributed by Hong Kong’s higher mortality rates in the 0, 25-59, and 60-74 age groups. In other words, higher male and female premature mortality rates in Hong Kong contributed to its higher life disparities. Over time, Hong Kong has achieved great improvements in reducing premature deaths, resulting in a significant fall in life disparity. Mortality decline at the 25-59 and the 60-74 age groups exerted great impacts on the decline in life disparity in Hong Kong. That was the main cause of the narrowing of the life disparity gap between Hong Kong and Japan.

For males, the period 1998-2016 deserves special attention and careful interpretation. It was a period when the gap in life disparity was almost closed, or sometimes, Hong Kong even had lower disparity compared with Japan. The absence of the gap was not because Hong Kong had exactly the same male mortality rates as Japan, rather, it was because Hong Kong had lower mortality rates across almost all age groups. That is, Hong Kong has outperformed Japan in male mortality in both the young and the old age groups. For females, the years 2005-2016 seemed to be a period when the JPN-HK gap in life disparity had been increasing again. However, it was actually driven by Hong Kong’s lower mortality at ages over 85. In other words, the mortality rate among the oldest elderly in Hong Kong has continued to improve and has maintained at a lower level than Japan, consequently enlarging the life disparity in Hong Kong. These findings again confirm the dominant impact of “saving lives at late ages” on increasing life disparity in the long-lived societies.

## Discussion and Conclusion

This study not only considers life expectancy, which is the fundamental statistic used to describe the mortality and health status, but also documents another dimension of mortality by analyzing life disparity, thus presenting a more comprehensive description of health conditions in Hong Kong. In addition, comparisons have been made with the trends in Japan, which has long held the life expectancy record since the mid-1970s,^23^ and has also remarkably succeeded in reducing life disparity.^7^ The comparison with Japan has enhanced the understanding of the health situation in Hong Kong in a global perspective.

This study found that between 1977 and 2016, in Hong Kong and Japan, life expectancy and life disparity presented a generally negative correlation in a long-term trend, which indicated that both Hong Kong and Japan could achieve higher life expectancies, and at the same time enjoy lower life disparities. The present findings were consistent with the results of Vaupel et al that found a strong negative correlation between the 2 dimensions of mortality.^7^

Besides, the decomposition analyses in the study have quantified the age-specific contributions to the improvement in life expectancy and the change in life disparity in the 2 societies. In both high-income societies, Hong Kong and Japan, the increase in life expectancy at this stage of development was mainly contributed by the reduction in mortality rates at the adult and old age groups, while the contributions from the infants, children and teenagers were rather minor. The decrease in life disparity was a net impact of the reduction in premature mortality, which offset the impact of the reduction in mortality among the oldest elderly.

Furthermore, this study has also evaluated the contributions of different ages to the overall gaps in life expectancy and life disparity between Hong Kong and Japan. The results show that although Hong Kong has achieved a higher level of life expectancy, life disparity in Hong Kong is still greater than Japan during the observed period. This comparison has revealed that the differences in life expectancy and life disparity between the 2 populations are closely related to their variations in age-specific mortality. Hong Kong has achieved great improvements in reducing premature deaths for both males and females, thus narrowing the gap in life expectancy and life disparity with Japan during the previous decades.

In addition, three findings should be singled out. First, in recent years, relative to Japan, the life expectancy advantages for both males and females in Hong Kong were driven by the dramatic decline in the mortality among the older population (85+ years), which on the other hand actually enlarged the life disparity gap with Japan. Second, the absence of the gap in male life disparity over recent years was not because the 2 societies had exactly the same male mortality rates but because Hong Kong had lower mortality across almost all age groups. Relative to Japan, Hong Kong’s lower premature mortality has reduced the gap, while its lower mortality after the threshold age has enlarged the gap. Third, the widening of the JPN-HK gap in female life disparity since 2005 was attributable to the impact of Hong Kong’s reduction in the mortality among the oldest elderly, which has more than offset the impact of its improvement in premature mortality. The JPN-HK comparison tells a different story from the US-UK comparison made by Shkolnikov and colleagues.^24^ In their paper, they compared the United States with England and Wales and found that the two countries had similar life expectancies, but the life expectancy losses were much greater in the United States because of the higher premature mortality.^24^ In contrast, the present study shows that Hong Kong is close to Japan in life expectancy, but has a higher life disparity because of Hong Kong’s lower mortality after the threshold ages. These 2 versions of the nexus between life expectancy and disparity highlight the need and significance of investigating the age-specific factors to unveil the underlying mortality dynamics.

This study has again confirmed that different levels of life disparity can be found in societies with similar levels of life expectancy.^1,25,26^ Based on life tables for 212 countries, Smits and Monden argued that countries that achieved a certain level of life expectancy earlier in time were more likely to experience higher levels of inequality.^1^ Nevertheless, the study conducted by Seaman and colleagues has shown some opposite findings: Scotland, which caught up with England and Wales in life expectancy, had higher lifespan variations, because of its lower older age mortality yet higher premature adult age mortality.^25^ Similarly, the JPN-HK comparison also shows that Hong Kong has reached the same level of life expectancy later in time as Japan but with higher life disparity, which is mainly due to the lower mortality at older ages in Hong Kong.

To some extent, the results on the rising role of older age mortality in shaping life disparity in Hong Kong indicate that the negative relationship between life expectancy and life disparity might turn into positive, and the 2 might increase at the same time. Such reversal is possible if deaths at old ages are further reduced or delayed, while mortality rates at younger age and midlife stall or start to rise.^26^ Previous studies have found the stagnation or increase in mortality at younger ages might be related with various socioeconomic factors, contributing to the differences in lifespan variation, particularly among some subpopulations.^27,28^ Therefore, policies related to the justification of social arrangements should be in place. In addition, Seligman et al revealed that the causes of death that led to greater life disparity were different from the causes that led to higher life expectancy, implying that promoting health at younger ages might increase equality while controlling cancer and cardiovascular diseases would help increase longevity.^29^ Therefore, it is very important to distribute healthcare resources to control the causes of death among both the young and the old, so that the society can achieve both equality and longevity.

The present findings also have some important policy implications. First, although the reduction in mortality among the oldest elderly leads to longer lifespan and higher disparity, it does not necessarily mean that they live healthily and happily at old ages. It would be even more meaningful to transform increased life expectancy to healthier and happier longevity.^30^ With more increasing elderly residents in Hong Kong and Japan, more attention should thus be paid to the medical treatment and healthcare needs of the aged. Second, to reduce the disparity in Hong Kong, further efforts should be made to improve the premature mortality. Targeted and effective health policy interventions are thus much needed to prevent premature deaths. Third, reduction in life disparity and rise in life expectancy have significant influence on the individuals’ consciousness of their own less-uncertain lifetimes and life plans,^7^ as well as implications on public policies. With the increasing chance to live a longer life, adjustments of retirement ages are suggested to keep in pace with the improvement in life expectancy. Also, the meanings of “old” and “dependent” may deserve reconsideration, especially in terms of the “prospective” measurements of ageing.^31^ For Hong Kong and Japan, in the context of both the highest life expectancies and ultra-low fertility rates in the world, extension of the retirement age might be a means to release human capital in the future.^31,32^

There are some limitations in this study. First, due to different sources of data, the smoothing methods for the life tables of Japan and Hong Kong as well as the open age groups are different, but that would only have a minor impact on the results. The robust check in the study shows the results do not change too much, and thus further justifies our conclusion. Second, due to the limitation of data, only age and gender were considered in the decomposition analyses, without looking into the causes of death. However, with access to more micro death registration data, the decomposition results can be developed to quantify the contributions of different diseases to the changes in life expectancy and life disparity. The age-, cause-specific analysis might provide more insights for public health policies. Third, this study focuses on the life expectancy and life disparity of the overall population, without exploring the patterns among different socioeconomic groups. Previous studies have revealed that life expectancy and lifespan variation can vary greatly across different socio-economic groups.^27,28,33^ Therefore, efforts can be made in future studies to address this aspect, which would be very meaningful. Fourth, this study does not investigate the impact of migration on the life expectancy and disparity. For Japan, compared to its total population, the number of international migrants is very small, which would have a very modest impact on its mortality rates. For Hong Kong, however, the number of immigrants (most of them from the Mainland China and other Asian countries) who are often at young ages, is non-negligible, which to some extent has lowered the mortality rates of the young population. This might partially explain why the mortality rates at the younger age groups in Hong Kong were lower than Japan in recent years (see Figure 1). Due to the limitation of data on migrants, the impact of migration cannot be quantified.

## Acknowledgments

The authors are grateful to the useful criticisms of the reviewers and the data made available by the Census and Statistics Department of the Hong Kong Government SAR.

## Ethical issues

Ethical approval was not required as the study was based on secondary data.

## Competing interests

Authors declare that they have no competing interests.

## Authors’ contributions

YZ conducted the study design, data collection, data analysis and wrote the manuscript; CM contributed to the data interpretation and revised the manuscript; PSFY contributed to the data interpretation, manuscript revision and supervised the study. All authors approved the final version of the manuscript.

## Funding

The research is supported by the RGC General Research Fund (106160261).

## Authors’ affiliations

^1^Department of Social Work and Social Administration, Faculty of Social Sciences, The University of Hong Kong, Hong Kong, China. ^2^Center for Demographic Research, Université catholique de Louvain, Ottignies-Louvain-la-Neuve, Belgium. ^3^HKJC Centre for Suicide Research and Prevention, The University of Hong Kong, Hong Kong, China.

## Supplementary files


Supplementary file 1 contains Figures S1-S2.

## Key Messages

**Implications for policy makers**

- Although Hong Kong has achieved a higher level of life expectancy, life disparity in Hong Kong is still greater than Japan during the observed period. Therefore, more policy concern is necessary to reduce the lifespan variability.
- Longer life expectancy and higher life disparity in Hong Kong were mainly attributable to the reduction in mortality among the oldest elderly for the study period.
- To reduce the life disparity in Hong Kong, efforts should be made to improve the premature mortality. Targeted and effective health policy interventions are thus much needed to prevent premature deaths.

**Implications for public**

The results suggest that different levels of life disparity can be found in societies with similar levels of life expectancy. If morality rates at old ages continue to decline while mortality rates at younger age and midlife level off or start to rise, then longevity and disparity may increase at the same time. It is therefore important to distribute healthcare resources to reduce deaths from both the young and the old, so that a society can achieve both longevity and health equality.
