# Supplementary material for: A Decomposition of Life Expectancy and Life Disparity: Comparison Between Hong Kong and Japan
Source: Int J Health Policy Manag. 2020 Jan 27;10(1):5–13. doi: 10.15171/ijhpm.2019.142 (PMC7947701; doi:10.15171/ijhpm.2019.142)
Supplement: Supplementary file 1 — contains Figures S1-S2. [file ijhpm-10-5-Supp1.pdf]

Supplementary file 1

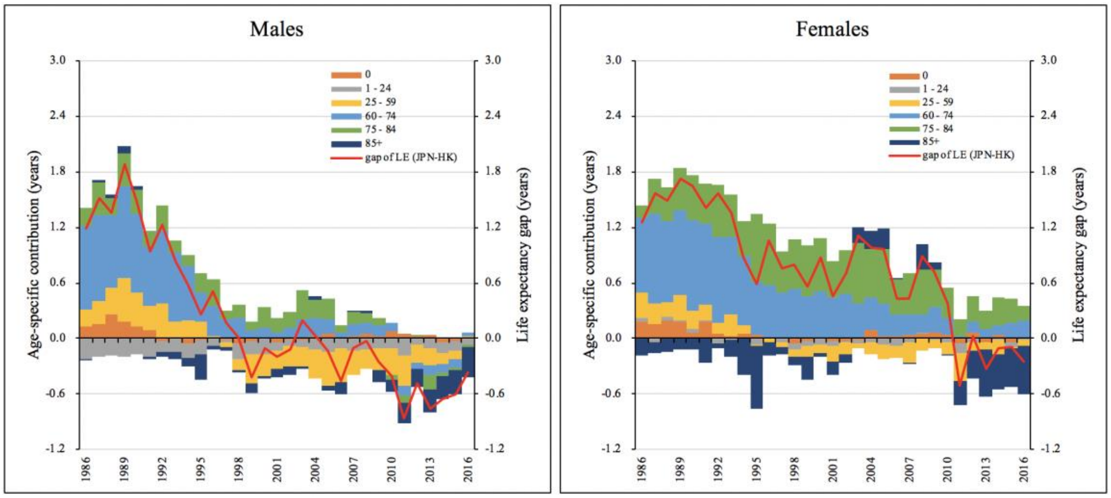

Figure S1. Decomposition of the gaps in life expectancy at birth between Japan and Hong Kong (HMD), 1986-2016. Note: red lines show the differences in life expectancy between Hong Kong and Japan (Japan minus Hong Kong, abbreviated as JPN-HK). The mortality data for Hong Kong were obtained from HMD. Due to the limitation of the data, the analysis was conducted over the period 1986-2016. Abbreviation: HMD, Human Mortality Database.

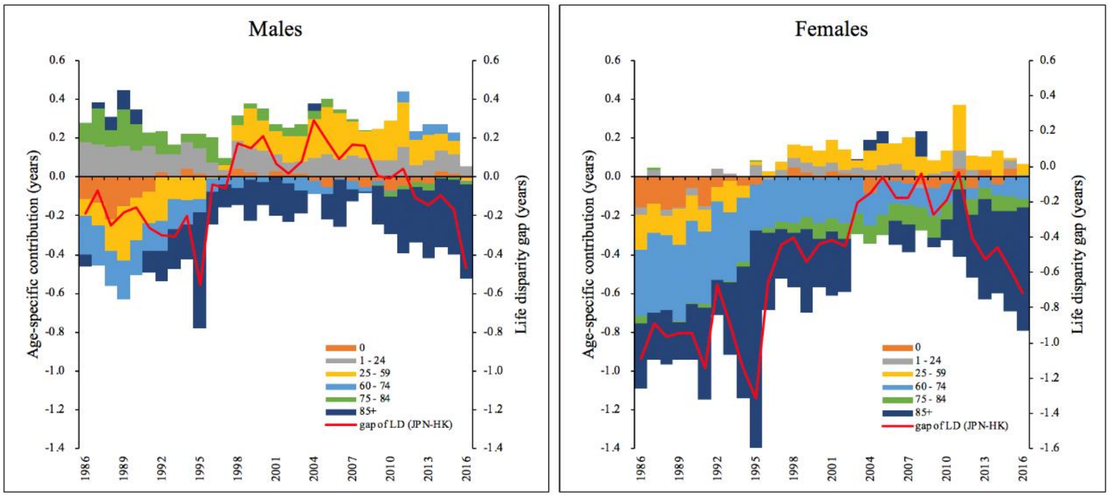

Figure S2. Decomposition of the gaps in life disparity at birth between Japan and Hong Kong (HMD), 1986-2016. Note: red lines show the differences in life disparity between Hong Kong and Japan (Japan minus Hong Kong, abbreviated as JPN-HK). The mortality data for Hong Kong were obtained from HMD. Due to the limitation of the data, the analysis was conducted over the period 1986-2016. Abbreviation: HMD, Human Mortality Database.
